# Supplementary material for: Predictors and outcome of first line treatment failure among under-five children with community acquired severe pneumonia at Bugando Medical Centre, Mwanza, Tanzania: A prospective cohort study
Source: PLoS One. 2020 Dec 11;15(12):e0243636. doi: 10.1371/journal.pone.0243636 (PMC7732094; doi:10.1371/journal.pone.0243636)
Supplement: Attachment — Submitted filename: Responses to reviewers.docx [file pone.0243636.s003.docx]

1. The two main issues raised by reviewer 2:

**a) To indicate deaths before 48h**

**Response:**

*No death before 48 hrs of follow up*

*Kindly Refer Study flow chart*

**b) Give the number excluded because of second line treatment**

**Response:**

*718 children not eligible for enrolment*

*•	416 children were out of study age limit*

*•	269 children had no features of SCAP*

*•	33 children had SCAP but were started on second line antibiotic treatment*

*Kindly Refer Study flow chart*

**2. A careful spelling check need to be addressed.**

**Response:**

*Spelling check was done.*

**3. Minor edits as per the other comments, including moving methods out of the results section**

**Response:**

*Summary of enrolment process was merged with participants’ characteristics*

**4. Considering the inclusion criteria for the prior studies discussed would also help improve the manuscript.**

**Response:**

*Thank you for this comment. The authors could not review the inclusion criteria since it would alter the current study findings, and may alter the conclusion drawn.*
